# Visual acuity and contrast sensitivity of adult zebrafish

**DOI:** 10.1186/1742-9994-9-10

**Published:** 2012-05-29

**Authors:** Christoph Tappeiner, Simon Gerber, Volker Enzmann, Jasmin Balmer, Anna Jazwinska, Markus Tschopp

**Affiliations:** 1 Department of Ophthalmology, University Hospital of Bern, Inselspital, Bern, Switzerland; 2 Department of Biology, University of Fribourg, Fribourg, Switzerland

**Keywords:** Adult zebrafish, Visual acuity, Optokinetic reflex, Optomotry

## Abstract

**Background:**

The aim of this study was to evaluate the visual acuity of adult zebrafish by assessing the optokinetic reflex. Using a modified commercially available optomotor device (OptoMotry®), virtual three-dimensional gratings of variable spatial frequency or contrast were presented to adult zebrafish. In a first experiment, visual acuity was evaluated by changing the spatial frequency at different angular velocities. Thereafter, contrast sensitivity was evaluated by changing the contrast level at different spatial frequencies.

**Results:**

At the different tested angular velocities (10, 15, 20, 25, and 30 d/s) and a contrast of 100%, visual acuity values ranged from 0.56 to 0.58 c/d. Contrast sensitivity measured at different spatial frequencies (0.011, 0.025, 0.5, 0.1, 0.2, 0.3, 0.4, 0.5 and 0.55 c/d) with an angular velocity of 10 d/s and 25 d/s revealed an inverted U-shaped contrast sensitivity curve. The highest mean contrast sensitivity (±SD) values of 20.49 ± 4.13 and 25.24 ± 8.89 were found for a spatial frequency of 0.05 c/d (angular velocity 10 d/s) and 0.1 c/d (angular velocity 25 d/s), respectively.

**Conclusions:**

Visual acuity and contrast sensitivity measurements in adult zebrafish with the OptoMotry® device are feasible and reveal a remarkably higher VA compared to larval zebrafish and mice.

## Background

Zebrafish (Danio rerio), which were initially used as an animal model in developmental biology due to high fecundity and ease of maintenance, has found its way into visual research due to its excellent visual system with a cone-dominated retina [1]. In the past, measurements of visual functions have been performed in larval zebrafish [2]. Furthermore, visual acuity (VA) of larval zebrafish has previously been determined by assessing the optokinetic reflex (OKR) in a self-constructed set-up [3]. Certain visual functions of adult zebrafish (gain of eye velocity at different spatial frequencies and contrast levels) have been published [4-6]. However, no empirical data about VA of adult zebrafish are available up to now. A standardized and reliable method for VA measurements is a prerequisite to analyze genetically modified fish lines and to evaluate the effect of a therapeutic action (e.g. impact of drugs or genetic interventions on the visual system) in addition to histological and ophthalmological examination of adult zebrafish [7]. OKR measurements have the advantage of a stereotyped and robust behavior and no prior training of the animal is necessary. The OKR consists of a slow movement in the direction of a perceived motion, followed by a quick return movement, called saccade [8]. Due to the relatively big eyes, the OKR of zebrafish can easily be detected. Therefore, the OptoMotry device was used for this study as it allows standardized OKR measurements.

OKR measurements are performed presenting moving gratings to the animal, and assessing reflexive responses, which are based on subcortical circuits. This technique allows to determine the spatial frequency threshold and constitutes one possibility to measure visual acuity. When referring to VA measurements based on OKR assessment, e.g. with the OptoMotry system, the term “visual acuity” corresponds to “spatial frequency threshold”. For simplicity and for the comparison to previous publications, only the term “visual acuity” is used in this manuscript.

The aim of the present study was to evaluate the VA and contrast sensitivity of adult zebrafish based on OKR assessments with a modified, commercially available and standardized system (OptoMotry).

## Materials and methods

Wild-type zebrafish (Danio rerio) of the AB (Oregon) strain, aged between 12 and 24 months, have been used. Experiments were performed at room temperature (approximately 20° Celsius). To facilitate the placement of the fish in the examination chamber, fish were shortly sedated in 0.1% Tricaine (Sigma-Aldrich, Buchs, Switzerland). Then, fish were irrigated with fresh water and were awake during the following experiment. Experimental research on animals has been approved by the governmental authorities and adhered to the ARVO Statement for the Use of Animals in Ophthalmic and Vision Research.

**Figure 1:**
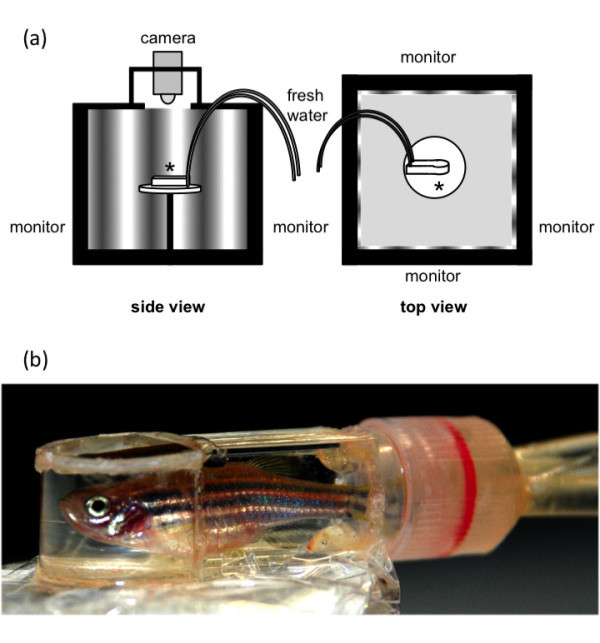
a) Schematic view of the OptoMotry device with the examination chamber (*) placed on the center of the OptoMotry platform (*adpated from Prusky et al.*[[9]**]). b)** The customized examination chamber enables a constant flow of fresh water and a broad visual field for the zebrafish.

The optokinetic stimulation was performed with a commercially available optomotor system designed for mice (OptoMotry®, CerebralMechanics, Lethbridge, AB, Canada) as previously described by *Prusky et al.*[9]*.* Virtual three-dimensional sine wave gratings were displayed on computer monitors, forming the walls of a cube (L x W x H = 39 x 39 x 32.5 cm; Figure 1a). The OptoMotry device was calibrated by centering platform location according to the manufacturer’s manual. The calibration was verified by manually measuring the distance between gratings and the distance from the platform to the monitors. In order to clearly see the eyes of the fish, optical zoom was applied to a final magnification of approximately 6x. Changing the zoom factor of the observing video system or varying the digital zoom in the software had no influence on the measurements after calibration.

The zebrafish was positioned in an examination chamber, which was designed to allow a constant flow of fresh water (Figure 1b). The hydrostatic pressure of an infusion bottle, placed 100 to 120 cm above the examination chamber, generated the necessary flow, which was adjustable by regulating the infusion valve. The examination chamber (L x W x H = 40 x 14 x 10 mm) was constructed with transparent polystyrene. The head of the zebrafish was located in the cylinder-shaped end of the chamber (R = 14 mm), allowing the animal an unhindered view to the sine wave gratings shown on the screens of the optomotor system. Inside the examination chamber, the animal was placed on a platform positioned 13 cm above the floor. Eye movements were recorded with a Sony DCR-HC26 Handycam®.

First, VA was determined with maximum contrast (100%) at varying angular velocities, namely 10, 15, 20, 25 and 30 degrees/second (d/s). At the beginning of the test, a low spatial frequency of 0.05 cycles/degree (c/d) was shown to the animal. Using the staircase strategy of the Optomotry device (OptoMotry version 1.7.0), the spatial frequency of the grating was increased until the animal no longer responded. Then, the spatial frequency was changed several times to identify the threshold. The response of the animal was assessed by three independent observers for the visual acuity experiment of adult zebrafish and by two independent observers for all other experiments. Judging the eye movements on a live video monitor (Additional file 1: Video S1), a correct response was defined as three or more consecutive saccades in the correct direction. Spatial frequencies higher than the threshold of the zebrafish result in random eye movements, similar to the random eye movement pattern observed with stationary gratings (Additional file 1: Video S1).

Afterwards, contrast sensitvitiy was evaluated by changing the contrast level (stair-case procedure) at different spatial frequencies (0.011, 0.025, 0.05, 0.1, 0.2, 0.3, 0.4, 0.5 and 0.55 c/d) until the zebrafish no longer responded with an OKR. Experiments were performed at an angular velocity of 10 and 25 d/s.

To compare results of the OptoMotry device to according data determined with another set-up [3], we have also measured larval zebrafish. A drop of water was placed on a plastic plate on the platform of the OptoMotry system. Then the larva was put into this drop of water. A Volk 2.2 lens was mounted 3.5 cm above the larva to allow an adequate magnification for the observation of the animals using the video system of the OptoMotry device (total magnification factor, including optical and digital zoom of the camera, of approximately 15x). An angular velocity of 15 d/s was chosen for direct comparison of the results to previously published data [3].

In order to extend VA measurements with the OptoMotry system to another species, also medaka (*Oryzias latipes),* aged 3 to 4 months, have been measured. An angular velocity of 15 and 25 c/d was chosen (contrast of 100%). The experimental procedure was analogue to the one in adult zebrfish. Since the size of adult zebrafish and medaka is similar, it was possible to use the same experimantation chamber.

The statistical analysis was performed using a Friedmann test with Dunns post-hoc analysis. A p-value < 0.05 was considered significant.

## Results

**Figure 2:**
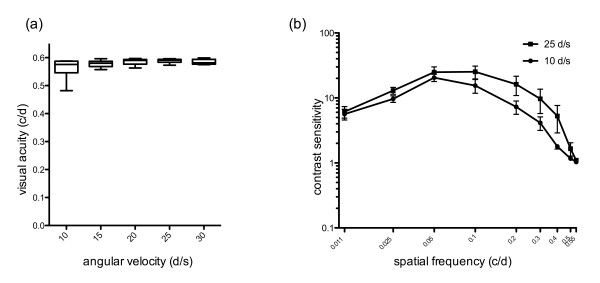
**a) All tested angular velocities (10, 15, 20, 25, 30 d/s) revealed similar visual acuity values (p > 0.05) at a contrast of 100% (n = 6 zebrafish for each tested angular velocity).** Box plots show the 25^th^, 50^th^, 75^th^ percentile; minimum and maximum for visual acuity values. **b)** Mean contrast sensitivity values measured at different spatial frequencies (0.011, 0.025, 0.05, 0.1, 0.2, 0.3, 0.4, 0.5 and 0.55 c/d) with an angular velocity of 10 d/s (n = 6 zebrafish) and 25 d/s (n = 6 zebrafish). The highest mean contrast sensitivity value was determined at a spatial frequency of 0.05 c/d for an angular velocity of 10 d/s and of 0.1 c/d for an angular velocity of 25 d/s, respectively. Whiskers indicate the 95% confidence interval (CI).

Using different angular velocities (10, 15, 20, 25, 30 d/s) with a constant contrast of 100%, no significant differences in mean VA values were found (Table 1, Figure 2a). The best mean VA of 0.589 ± 0.019 c/d was achieved with an angular velocity of 25 d/s. VA was slightly lower (p > 0.05) with the higher and lower tested angular velocities, revealing the lowest mean VA of 0.563 c/d at an angular velocity of 10 d/s. Standard deviation (SD 0.032 c/d) was significantly higher at the lowest tested angular velocity of 10 d/s, and was comparably low at all other velocities (15 d/s: SD = 0.021; 20 d/s: SD = 0.016; 25 d/s: SD = 0.015; 30 d/s SD = 0.019).

**Table 1:** Mean visual acuity and standard deviation (SD) for different angular velocities in adult zebrafish (3 experiments with independent observers and n = 6 zebrafish, each) at a contrast of 100%

**Angular velocity (d/s)**	**Mean visual acuity ± SD (c/d)**
**10**	0.563 ± 0.032
**15**	0.578 ± 0.021
**20**	0.586 ± 0.016
**25**	0.589 ± 0.015
**30**	0.584 ± 0.019

Determining contrast sensitivity at different spatial frequencies (0.011, 0.025, 0.05, 0.1, 0.2, 0.3, 0.4, 0.5 and 0.55 c/d) revealed an inverted U-shaped contrast sensitivity curve (Figure 2b). Mean contrast sensitivity values and their standard deviation are shown in Table 2. The highest mean contrast sensitivity values of 20.49 ± 4.13 and 25.24 ± 8.89 were found for a spatial frequency of 0.05 c/d (angular velocity 10 d/s) and 0.1 c/d (angular velocity 25 d/s), respectively.

**Table 2:** Mean contrast sensitivity and standard deviation (SD) for tested spatial frequencies (0.011 – 0.55 c/d) at two different angular velocities (10 d/s and 25 d/s) (2 experiments with independent observers and n=6 zebrafish, each)

	**Contrast sensitivity**	
**Spatial frequency (c/d)**	**10 d/s**	**25 d/s**
0.011	5.63 ± 1.66	6.12 ± 1.94
0.025	9.66 ± 1.75	12.97 ± 2.47
0.05	20.49 ± 4.13	24.91 ± 8.08
0.1	15.51 ± 5.83	25.24 ± 8.89
0.2	7.28 ± 2.64	16.24 ± 8.41
0.3	4.14 ± 1.53	9.70 ± 6.19
0.4	1.79 ± 0.24	5.27 ± 3.74
0.5	1.17 ± 0.11	1.66 ± 0.60
0.55	1.04 ± 0.04	1.10 ± 0.11

For larval zebrafish, a mean VA of 0.16 ± 0.016 c/d was determined at an angular velocity of 15 d/s and a contrast of 100%. Adult medaka revealed a mean VA of 0.53 ± 0.054 c/d at an angular velocity of 15 d/s and and a VA of 0.56 ± 0.045 c/d at an angular velocity of 25 d/s (contrast of 100%, each).

## Discussion

Visual acuity, defined as the ability to distinguish two objects (spatial resolution) at a given angular distance, is limited by different factors of the visual system, e.g. dimensional and optical properties of the eye, neural properties of the retina and visual processing in higher brain centers. Particularly, the distance between photoreceptor cells plays a pivotal role [3]. Based on photoreceptor spacing and optical properties of the eye, Haug et al. calculated a maximum possible VA of 0.24 c/d in 5-day old larval zebrafish and of 0.871 c/d in adult zebrafish [3]. This difference in VA is mainly explained by the different eye size: due to geometrical reasons a larger eye leads to a larger retinal image with better VA as photoreceptor spacing is comparable between the two development stages [3].

Most VA measurement techniques available for animal experiments [10-14] require prior training of the animal and are time-consuming. In contrast, VA testing methods based on an innate behavior need no prior training of the animal. In mice and rat, VA can be measured using the optomotor reflex [9]. Briefly, a virtual rotating cylinder with vertical sine wave grating provokes reflexive head and neck movements. Like the optomotor reflex, also the OKR is based on innate reflexive behavior. The OKR can be repeatedly provoked independently of the animal’s cooperation and their large eyes can easily be observed, which makes assessment of the OKR an ideal method for VA measurements in zebrafish.

As larval zebrafish absorb enough oxygen through their skin, they can be embedded in methylcellulose for VA measurements [3]. This approach is not feasible in adult zebrafish, which need a constant flow of oxygenated water irrigating their gills. Restricting body movements in the adult zebrafish was rather challenging, we did however solve this problem by designing a custom made flow through chamber. It was not necessary to fixate the body of the fish in our experiments, as the small chamber offered enough restriction to allow the observation of eye movements. This is likely less incriminating to the fish. However, a fixation would still be possible with the provided set-up (for instance by clamping the body of the anesthetized fish between two pieces of sponge [4]) if necessary for certain experiments.

With optimal settings, we determined a best mean VA of 0.59 c/d in adult zebrafish and a best mean VA of 0.16 c/d in larval zebrafish. The latter is equal to the mean VA of larval zebrafish in a previous study using another set-up [3]. The VA of adult zebrafish is higher than the mean VA measured in larval zebrafish but lower than the mathematically one estimated by Haug et al. [3]. The ratio of the measured and the calculated VA was similar for larval and adult zebrafish (0.67 for larval and 0.68 for adult zebrafish). It may be assumed that the theoretically possible VA in the mathematical model is not achievable due to optical imperfections. On the other hand, VA values also depend on measurement techniques, e.g., in humans the VA obtained by subjective testing with a visual acuity chart is known to be higher than the one obtained by OKR measurements [15,16].

To exemplarily demonstrate that the OptoMotry device can also be used to determine VA in other species, we have tested medaka with the same standardized set-up. The adult fish of both species, zebrafish and medaka, revealed a comparable VA.

Contrast sensitivity is an important parameter of vision. In humans, the impairment of contrast sensitivity can precede loss of visual acuity and is related to functional disability [17]. The relation of contrast sensitivity and spatial frequency can be depicted as a contrast sensitivity curve. In adult zebrafish a typical inverted U-shaped contrast sensitivity curve with a fall off in sensitivity at low and high spatial frequencies can be found which is also seen in other species including humans [9,18,19].

A similar peak contrast sensitivity as in adult zebrafish has been found in adult mice, also using the OptoMotry device [9]. In humans, contrast sensitivity is significantly higher [17]. On the other hand, contrast sensitivity of adult zebrafish is considerably higher than in larval zebrafish [20] and birds [21]. Overall, adult zebrafish reveal quite a good contrast sensitivity, which might be an adaption to their natural habitat of shallow waterbodies with silty substrate [22].

## Conclusions

Measurement of VA and contrast sensitivity based on the OKR is feasible in adult zebrafish, revealing a remarkable higher VA compared to larval zebrafish and mice [3,12]. As the OptoMotry device is commercially available, standardized and reproducible measurements are possible. We believe that the presented set-up does not only allow screening for adult vision mutants but will also help to better analyze the vision of wild-type or mutant zebrafish and pharmacologically induced disease models.

## Abbreviations

VA, Visual acuity; OKR, Optokinetic reflex; OMR, Optomotor reflex.

## Competing interests

The authors declare that they have no competing interests.

## Authors’ contributions

MT conceived of the study and designed the experiments. CT performed the statistical analysis and wrote the manuscript. CT, VE and AJ were involved in the discussion of the study design. MT, SG, JB and CT performed the experiments. MT, SG and VE contributed to writing of the manuscript. All authors contributed to and approved the final manuscript.

## Supplementary Material

Additional file 1 **Video S1.** Top view of an adult zebrafish in the examination chamber displaying the optokinetic reflex (OKR), which consists of a slow movement in the direction of a perceived motion, followed by a quick return movement. The direction of the OKR can be easily assessed. First, stationary gratings are presented to the fish, followed by well visible moving gratings (spatial frequency 0.05 c/d; contrast 100%; angular velocity 30 d/s). At the end, stationary gratings are again presented to the fish. The red stripes have been added to the video to simulate the direction of the grating. Click here for file

## References

[B1] Fadool JM Dowling JE Zebrafish: a model system for the study of eye genetics Prog Retin Eye Res 2008 27 89 110 10.1016/j.preteyeres.2007.08.002 17962065 PMC2271117

[B2] Beck JC Gilland E Tank DW Baker R Quantifying the ontogeny of optokinetic and vestibuloocular behaviors in zebrafish, medaka, and goldfish J Neurophysiol 2004 92 3546 3561 10.1152/jn.00311.2004 15269231

[B3] Haug MF Biehlmaier O Mueller KP Neuhauss SC Visual acuity in larval zebrafish: behavior and histology Front Zool 2010 7 8 10.1186/1742-9994-7-8 20193078 PMC2848032

[B4] Mueller KP Schnaedelbach ODR Russig HD Neuhauss SCF VisioTracker, an innovative automated approach to oculomotor analysis. J Vis Exp 2011 10.3791/3556 10.3791/3556 PMC3227217 22005608

[B5] Zou S-Q Yin W Zhang M-J Hu C-R Huang Y-B Hu B Using the optokinetic response to study visual function of zebrafish. J Vis Exp 2010 10.3791/1742 10.3791/1742 PMC2818711 20125082

[B6] Mueller KP Neuhauss SCF Quantitative measurements of the optokinetic response in adult fish J Neurosci Methods 2010 186 29 34 10.1016/j.jneumeth.2009.10.020 19900474

[B7] Tschopp M Takamiya M Cerveny KL Gestri G Biehlmaier O Wilson SW Strähle U Neuhauss SCF Funduscopy in adult zebrafish and its application to isolate mutant strains with ocular defects PLoS One 2010 5 e15427 10.1371/journal.pone.0015427 21079775 PMC2974646

[B8] Huang Y-Y Neuhauss SCF The optokinetic response in zebrafish and its applications Front Biosci 2008 13 1899 1916 10.2741/2810 17981678

[B9] Prusky GT Rapid quantification of adult and developing mouse spatial vision using a virtual optomotor system Invest Ophthalmol Vis Sci 2004 45 4611 4616 10.1167/iovs.04-0541 15557474

[B10] Carlsson MA Swedberg MDB A behavioural operant discrimination model for assessment and pharmacological manipulation of visual function in rats Brain Res 2010 1321 78 87 20097183 10.1016/j.brainres.2010.01.029

[B11] Macuda T Gegear RJ Laverty TM Timney B Behavioural assessment of visual acuity in bumblebees (Bombus impatiens) J Exp Biol 2001 204 559 564 11171306 10.1242/jeb.204.3.559

[B12] Prusky GT West PW Douglas RM Behavioral assessment of visual acuity in mice and rats Vision Res 2000 40 2201 2209 10.1016/S0042-6989(00)00081-X 10878281

[B13] Seymoure P Juraska JM Vernier and grating acuity in adult hooded rats: the influence of sex Behav Neurosci 1997 111 792 800 9267656 10.1037//0735-7044.111.4.792

[B14] Thomas BB Samant DM Seiler MJ Aramant RB Sheikholeslami S Zhang K Chen Z Sadda SR Behavioral evaluation of visual function of rats using a visual discrimination apparatus Journal of Neuroscience Methods 2007 162 84 90 10.1016/j.jneumeth.2006.12.010 17289151 PMC3074943

[B15] Han SB Han ER Hyon JY Seo J-M Lee JH Hwang J-M Measurement of distance objective visual acuity with the computerized optokinetic nystagmus test in patients with ocular diseases Graefes Arch Clin Exp Ophthalmol 2011 249 1379 1385 10.1007/s00417-011-1705-x 21603927

[B16] Hyon JY Yeo HE Seo J-M Lee IB Lee JH Hwang J-M Objective measurement of distance visual acuity determined by computerized optokinetic nystagmus test Invest Ophthalmol Vis Sci 2010 51 752 757 10.1167/iovs.09-4362 19834033

[B17] Owsley C Contrast sensitivity Ophthalmol Clin North Am 2003 16 171 177 10.1016/S0896-1549(03)00003-8 12809156

[B18] Haughom B Strand T-E Sine wave mesopic contrast sensitivity - defining the normal range in a young population 2011 1755 3768 10.1111/j.1755-3768.2011.02323.x 10.1111/j.1755-3768.2011.02323.x 22176733

[B19] Hagemans KH van der Wildt GJ The influence of the stimulus width on the contrast sensitivity function in amblyopia Invest Ophthalmol Vis Sci 1979 18 842 847 457357

[B20] Rinner O Contrast sensitivity, spatial and temporal tuning of the larval zebrafish optokinetic response Invest Ophthalmol Vis Sci 2005 46 137 142 10.1167/iovs.04-0682 15623766

[B21] Ghim MM Hodos W Spatial contrast sensitivity of birds J Comp Physiol A Neuroethol Sens Neural Behav Physiol 2006 192 523 534 10.1007/s00359-005-0090-5 16404602

[B22] Spence R Gerlach G Lawrence C Smith C The behaviour and ecology of the zebrafish, Danio rerio Biol Rev Camb Philos Soc 2008 83 13 34 18093234 10.1111/j.1469-185X.2007.00030.x

